# Rotational femoral osteotomies and cam resection improve hip function and internal rotation for patients with anterior hip impingement and decreased femoral version

**DOI:** 10.1093/jhps/hnad018

**Published:** 2023-07-26

**Authors:** Till D Lerch, Malin K Meier, Markus. S Hanke, Adam Boschung, Florian Schmaranzer, Klaus A Siebenrock, Moritz Tannast, Simon D Steppacher

**Affiliations:** Department of Orthopaedic Surgery, Inselspital, Bern University Hospital, University of Bern, Freiburgstrasse 10, Bern 3010, Switzerland; Department of Diagnostic, Interventional and Paediatric Radiology, University of Bern, Inselspital, Bern University Hospital, Freiburgstrasse 10, Bern, 3010, Switzerland; Department of Orthopaedic Surgery, Inselspital, Bern University Hospital, University of Bern, Freiburgstrasse 10, Bern 3010, Switzerland; Department of Orthopaedic Surgery, Inselspital, Bern University Hospital, University of Bern, Freiburgstrasse 10, Bern 3010, Switzerland; Department of Orthopaedic Surgery, Inselspital, Bern University Hospital, University of Bern, Freiburgstrasse 10, Bern 3010, Switzerland; Department of Orthopaedic Surgery, Inselspital, Bern University Hospital, University of Bern, Freiburgstrasse 10, Bern 3010, Switzerland; Department of Diagnostic, Interventional and Paediatric Radiology, University of Bern, Inselspital, Bern University Hospital, Freiburgstrasse 10, Bern, 3010, Switzerland; Department of Orthopaedic Surgery, Inselspital, Bern University Hospital, University of Bern, Freiburgstrasse 10, Bern 3010, Switzerland; Department of Orthopaedic Surgery, Inselspital, Bern University Hospital, University of Bern, Freiburgstrasse 10, Bern 3010, Switzerland; Department of Orthopaedic Surgery and Traumatology, Fribourg Cantonal Hospital, University of Fribourg, Freiburg 3010, Switzerland; Department of Orthopaedic Surgery, Inselspital, Bern University Hospital, University of Bern, Freiburgstrasse 10, Bern 3010, Switzerland

## Abstract

Femoroacetabular impingement (FAI) patients with reduced femoral version (FV) are poorly understood. The aim of this study is to assess (i) hip pain and range of motion, (ii) subjective satisfaction and (iii) subsequent surgeries of symptomatic patients who underwent rotational femoral osteotomies. A retrospective case series involving 18 patients (23 hips, 2014–2018) with anterior hip pain that underwent rotational femoral osteotomies for treatment of decreased FV was performed. The mean preoperative age was 25 ± 6 years (57% male), and all patients had decreased FV < 10° and minimum 1-year follow-up (mean follow-up 2 ± 1 years). Surgical indication was the positive anterior impingement test, limited internal rotation (IR) in 90° of flexion (mean 10 ± 8°) and IR in extension (mean 24 ± 11°), anterosuperior chondrolabral damage in Magnet resonance (MR) arthrography, CT-based measurement of decreased FV (mean 5 ± 3°, Murphy method) and no osteoarthritis (Tönnis Grade 0). Most patients had intra- and extra-articular subspine FAI (patient-specific 3D impingement simulation). Subtrochanteric rotational femoral osteotomies to increase FV (correction 20 ± 4°) were combined with cam resection (78%) and surgical hip dislocation (91%). (i) The positive anterior impingement test decreased significantly (*P* < 0.001) from pre- to postoperatively (100% to 9%). IR in 90° of flexion increased significantly (*P* < 0.001, 10 ± 8° to 31 ± 10°). (ii) Subjective satisfaction increased significantly (*P* < 0.001) from pre- to postoperatively (33% 77%). The mean Merle d’Aubigné and Postel score increased significantly (*P* < 0.001) from 14 ± 2 (8–15) points to 17 ± 1 (13–18, *P* < 0.001) points. Most patients (85%) reported at follow-up that they would undergo surgery again. (iii) At follow-up, all 23 hips were preserved (no conversion to total hip arthroplasty). One hip (4%) underwent revision osteosynthesis. Proximal rotational femoral osteotomies combined with cam resection improve hip pain and IR in most FAI patients with decreased FV at short-term follow-up. Rotational femoral osteotomies to increase FV are safe and effective.

## INTRODUCTION

Femoroacetabular impingement (FAI) is increasingly recognized as a cause of hip pain and early-onset osteoarthritis in young and active patients [1]. Cam, pincer and mixed-type FAI deformities were described as causes for anterior hip impingement by Ganz *et al*. in 2003 [2]. By then, variations of femoral version (FV) such as increased or decreased FV were not taken into consideration. Meanwhile, the influence of both decreased FV and increased FV for patient-related outcomes after hip arthroscopy for FAI [3] was investigated. Previously, decreased FV was associated with slipped capital femoral epiphysis (SCFE) [4]. More recently, decreased FV was associated with revision hip surgery after hip arthroscopy at a 2-year follow-up [5]. In addition, variations of FV were investigated in patients with FAI [6]. Decreased FV (<10°) was found in 22% of young patients with hip pain due to FAI or hip dysplasia [6]. Elevated FV was associated with hip dysplasia [7] and posterior hip impingement [8]. Previous studies observed an association between abnormal FV and the development of osteoarthritis of the hip [9].

Recent studies investigated the clinical significance of FV for FAI patients and for hip preservation surgery [10–12]. Inferior clinical outcomes after hip arthroscopy [12] in patients with increased FV have been observed. At the same time, posterior extra-articular hip impingement and increased FV were observed as contributing to hip pain [8, 13]. On the other hand, some authors recognized decreased FV (called excessive femoral retroversion) as a relative contraindication for FAI surgery because it was associated with poor outcomes after hip arthroscopy for FAI [3]. Treatment is discussed controversially because some authors reported that FV does not influence the outcomes after hip arthroscopy for FAI [14], while others reported poor outcomes after hip arthroscopy [3]. To date, FAI patients with decreased FV are poorly understood. Recently, anterior extra-articular subspine impingement was observed in patients with decreased FV combined with a cam-type morphology [15]. Reduced hip internal rotation (IR) was recognized in cam-type FAI, typically in 90° of flexion [13, 16]. Decreased FV also reduces IR [9] and therefore could contribute to anterior FAI [9, 17]. In 1991, the so-called diminished femoral antetorsion syndrome was described [17] and rotational osteotomies were performed thereafter in adult and adolescent patients [9, 18] to increase FV and to reduce hip pain and osteoarthritis. More recently, similar outcomes were reported in a small matched cohort study comparing hip arthroscopy and femoral rotation osteotomy to treat patient with decreased FV [19].

The purpose of this study was to assess (i) hip pain, range of motion (ROM) and function, (ii) subjective satisfaction and (iii) subsequent surgeries in patients with symptomatic anterior intra- and extra-articular subspine FAI and decreased FV that underwent rotational subtrochanteric femoral osteotomies.

## Materials AND METHODS

This is an IRB (institutional review board)-approved retrospective case series of 18 patients (23 hips, 2014–18) with anterior FAI and decreased FV that underwent rotational subtrochanteric femoral osteotomies to increase FV. The mean age of the patients was 25 ± 6 years (57% male) with a minimum 1-year follow-up (mean follow-up time 2 years, range 1–5 years, Table I).

**Table I.** Demographic data of the patient series are shown

*Parameter*	*Value*
Total hips	23 (18)
Age at operation (years)	25 ± 6 (15–38)
Gender (% male of all hips)	57
Side (% right of all hips)	52
Height (cm)	176 ± 10 (162–192)
Weight (kg)	76 ± 22 (49–131)
Body mass index (kg/m^2^)	24 ± 6 (17–36)
Follow-up time (years)	2 ± 1 (1–5)
Intraoperative surgical correction (°)	20 ± 4 (15–30)
Hips (%) with SHD	21 (91)
Dynamic compression plate (DCP) 6-hole steel plate for fixation [number (% of all hips)]	22 (96)
Paediatric hip plate for fixation [number (% of all hips)]	1 (4)
Hips (%) with previous surgeries	9 (39)
Hips (%) with previous hip arthroscopies for treatment of anterior FAI	5 (22)

Values are expressed as mean ± SD and range in parentheses.

### Patient selection

**Fig. 1.**
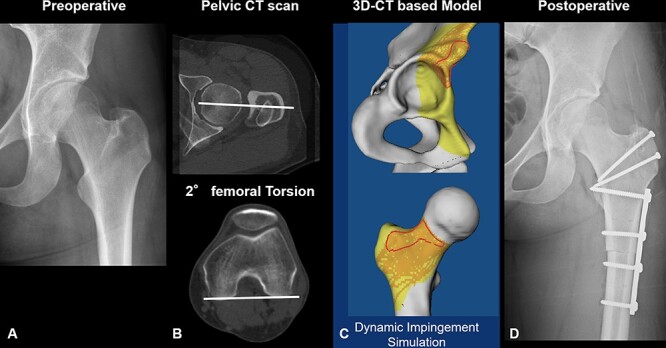
Preoperative AP radiograph (A), measurement of FV (B) and 3D-CT-based model of the pelvis and the proximal femur (C) showing anterior intra- and extraarticular hip impingement conflict of a 20-year-old male patient who underwent subtrochanteric rotational femoral osteotomy to increase FV combined with SHD (D).

Patients who underwent rotational subtrochanteric femoral osteotomies (Fig. 1) to increase FV between January 2014 and December 2018 were included and retrospectively reviewed. Exclusion criteria were any type of previous hip surgery altering FV (e.g. previous femoral osteotomy), a concomitant valgus deformity treated with an intertrochanteric varus osteotomy, post-traumatic deformity, patients with cerebral palsy, skeletally immature patients (stage < 4 according to Risser staging), avascular necrosis of the femoral head or sequalae of Legg-Calve-Perthes disease. One patient had unilateral SCFE in childhood and underwent bilateral rotational subtrochanteric femoral osteotomy and was included.

### Clinical diagnosis and evaluation

All patients were clinically examined by one of our attending hip surgeons with expertise in hip preservation surgery (blinded). This included a thorough acquisition of the patient history, a goniometric measurement of the hip range of motion (ROM, Table II) and the evaluation of the anterior and posterior impingement tests [16]. The anterior impingement test (flexion, adduction and internal rotation, FADIR test) was assessed preoperatively and postoperatively in all patients (Table II) and was considered positive, if the patient exhibited pain in 90° of flexion combined with forced IR and adduction [16]. Diagnostic intra-articular injections of steroid or local anaesthesia were performed in some of the patients.

**Table II.** Clinical results and ROM values of the patient series are shown

*Parameter*	*Preoperative*	*At follow-up*	*P-value preoperative versus follow-up*
MDA score (18–0) [31]	14 ± 2 (8–15)	17 ± 1 (13–18)^*^	<0.001
Anterior impingement test (% of hips) [16]	100	9^*^	<0.001
Subjective hip value	33 ± 25 (0–75)	77 ± 16 (50–100)^*^	<0.001
ROM (°)			
Flexion	97 ± 10 (80–120)	106 ± 12 (90–120)^*^	0.003
IR in 90° of flexion	10 ± 8 (0–30)	31 ± 10 (15–50)^*^	<0.001
External rotation in 90° of flexion	54 ± 18 (20–80)	38 ± 12 (20–60)^*^	0.006
Abduction in extension	42 ± 9 (30–50)	35 ± 13 (20–45)	0.179
Adduction in extension	23 ± 5 (20–30)	23 ± 6 (20–30)	0.485
IR in extension	24 ± 11 (0–45)	36 ± 10 (20–50)	0.593
External rotation in extension	49 ± 9 (40–70)	41 ± 8 (30–60)	0.625
WOMAC	n/a	17 ± 14 (0–48)	
UCLA score	n/a	6 ± 2 (3–10)	

Continuous values are expressed as mean ± SD and range in parentheses. WOMAC=Western Ontario and McMaster Universities Osteoarthritis Index. UCLA= University of California at Los Angeles Activity score. MDA= Merle d’Aubigne score.

* Significant difference compared to the preoperative value.

Surgical indication to perform rotational osteotomy was a positive anterior impingement test, limited IR in 90° of flexion (mean 10 ± 8°, Table II) and IR in extension (mean 24 ± 11°, Table II), anterosuperior chondrolabral damage in MR arthrography and CT-based measurement of abnormal low FV (mean FV 5 ± 3°, Table III). Surgery was only performed in patients with symptoms lasting at least 6 months and if the patient had failed all nonsurgical treatments including physical therapy. Surgery was performed if the ROM abnormality correlated with the decreased FV in prone position. This means that decreased hip IR was combined with decreased FV. Some patients reported pain during sports, while most reported pain during daily activities such as sitting or driving a car. Diagnosis of anterior extra-articular subspine hip impingement was based on the combination of clinical (positive anterior impingement test) and radiographic findings (FV on CT scan), as well as dynamic impingement conflict during 3D dynamic impingement simulation [15] (Fig. 1C).

**Table III.** Preoperative radiographic information of the patient series is shown

*Parameter*	*Value*
Alpha angle AP (°)	50 ± 11 (38–75)
Alpha angle on lateral view (°)	48 ± 8 (37–63)
LCE angle (°)	31 ± 9 (16–49)
Neck-shaft angle (°)	130 ± 5 (120–138)
Acetabular index (°)	3 ± 4 (−6–10)
Extrusion index (°)	21 ± 5 (14–29)
CT-based measurements	
FV, Murphy method (°)	5 ± 3 (−1–9)
Central acetabular version (°)	14 ± 6 (3–25)
Combined version, McKibbin index (°)	19 ± 7 (14–24)

Continuous values are expressed as mean ± SD and range in parentheses. LCE=lateral center edge angle. FV=femoral version, also called femoral torsion or femoral antetorsion.

### Radiographic evaluation

Routine radiographic evaluation generally consisted of an anteroposterior (AP) pelvic radiograph taken using a standardized technique [16] and a cross-table lateral radiograph of the hip. All patients had no osteoarthritic changes (Tönnis Grade 0) on preoperative AP pelvic views. The AP pelvic radiograph was then assessed using previously described and validated computer software (Hip^2^Norm, University of Bern, Bern, Switzerland) [20] for measurement of eight radiographic parameters of the hip joint with high accuracy and reliability. The alpha angle was measured on the axial cross-table radiograph (Table III) to assess asphericity of the femoral head. All radiographic measurements were performed by two independent observers (blinded). The analysis revealed concomitant cam deformities in 18 hips (78%), while five hips had pincer deformity (lateral center edge LCE angle >39°). Four hips had severe acetabular retroversion that underwent concomitant anteverting periacetabular osteotomy (PAO). One patient had a symptomatic hip dysplasia, which was treated with a concomitant PAO.

All symptomatic patients underwent MR arthrography and CT of the pelvis. FV was measured on CT scans (Fig. 1B) according to the method described by Murphy [6, 21]. The Murphy method uses two images for the proximal femur: first, the centre of the femoral head and a second CT image through the base of the femoral neck [21]. This showed better reproducibility (a variance of 0.4° and a standard deviation of 0.6°) compared to one single transverse CT section through the femoral neck [22]. All patients had decreased FV [9] (defined as below 10° of FV), in accordance with previous studies [6, 17]. The mean FV of all patients was 5 ± 3° (−1–9, Table III).

All patients underwent magnetic resonance imaging (MRI) arthrography [23] of the hip according to a standardized technique [24]. In brief, the scans were performed using a Siemens TRIO 3.0T high-field scanner (Erlangen, Germany) with a flexible surface coil after fluoroscopic-guided arthrography. MRI was performed to detect intra-articular lesions.

CT scans were acquired according to a previously validated protocol [15, 25] and were used for three-dimensional (3D) virtual simulation of hip motion and impingement analysis [25].

Nine hips (39%) had previous unsuccessful hip-preserving surgery. Previous unsuccessful HA was performed in five hips (22%, Table I).

### Patient-specific 3D simulation of hip impingement

Segmentation of a 3D model of the osseous pelvis and the femur (Fig. 1C) using the Amira Visualization Toolkit (Visage Imaging Inc, Carlsbad, CA, USA) was performed for all patients. Using this 3D model based on the CT scans, the simulated ROM and the individual impingement were analysed for all patients (Fig. 1C) using a collision detection software.

CT-based 3D models were evaluated using previously described and validated software [25]. This software uses automatic detection of the acetabular rim [26], a best-fitting sphere algorithm to localize the femoral head centre and the equidistant method for ROM analysis [27]. In brief, the anterior impingement test [16] was simulated as previously described. IR was calculated in 90° of flexion (Fig. 1C) combined with 0°, 10°, 20° and 30° of adduction (Table IV) as well as in isolated 90° of flexion. Location of impingement was displayed using previously used clock system [15] with 6 o’clock representing the acetabular notch. Three o’clock position was consistently defined anteriorly independent of the side (for both right and left hips). In addition, the patient-specific extra- or intra-articular impingement location was analysed. Intra-articular locations comprised the acetabular rim and the lunate surface [24] (acetabular side) and the femoral head and neck (femoral side). All patients (100%, Table IV, Supplementary Fig. S1) had intra-articular hip impingement, and most had extra-articular subspine FAI (55–85%) evaluating the anterior impingement test with 20° and 30° adduction using patient-specific 3D simulation.

**Table IV.** Frequency of anterior intra- and extra-articular (subspine) hip impingement during the anterior impingement test of the dynamic 3D CT-based impingement simulation is shown

*Test*	*Anterior intra-articular impingement (%)*	*Anterior subspine extra-articular impingement (%)*
Anterior impingement test^a^	75	20
Anterior impingement test^a^ with 10° adduction	95	35
Anterior impingement test^a^ with 20° adduction	100	55
Anterior impingement test^a^ with 30° adduction	100	85

a Anterior impingement test signifies 90° of flexion and 30° of IR.

### Surgical technique

Rotational femoral osteotomies to increase FV were combined with a surgical hip dislocation (SHD) in 91%. SHD was added for correction of cam deformities (78%) and/or treatment of labrum lesions (Fig. 1). The mean intraoperative correction of FV was 20 ± 4° (Table I).

SHD allows comprehensive evaluation of hip ROM and intra-/extra-articular impingement and visualization of the entire joint. Typically, surgery started with SHD for the following reasons: (i) assessment of intra- and extra-articular anterior FAI during execution of the anterior impingement test, (ii) correction of the intra-articular osseous conflict, (iii) evaluation and potential repair of acetabular chondrolabral damage, (iv) evaluation of anterior impingement conflict after cam resection and/or acetabular rim trimming and (v) judgement of an additional anterior resection of the greater trochanter.

### Correction of FV

Intraoperative correction of FV was evaluated using K wires (positioned in the proximal and distal fragment), which were inserted before the femoral osteotomy [9, 28]. Mostly 20° (and maximal 30° for one patient) of intraoperative correction was planned to avoid overcorrection, depending on the preoperative value of FV. No particular range of postoperative FV was aimed for. Compression was usually applied before fixation of the subtrochanteric femoral osteotomy. If IR was insufficient after cam resection, femoral osteotomy was performed. Repeated intraoperative evaluation of IR was performed. In the presence of a pre-existing cam deformity (alpha angle >50° [29]), cam resection was performed before the femoral osteotomy. An anterior resection of the greater trochanter was evaluated before the femoral osteotomy in the case of extra-articular FAI. The anterior resection of the greater trochanter was thus a potential additive step during this procedure. We expected that rotational osteotomies can lead to increased IR and decreased hip external rotation. The intraoperative surgical goal was to achieve or to maintain 30° of IR in 90° of hip flexion. No particular range of FV was the intraoperative goal.

### Evaluation at follow-up

All patients were contacted by phone, were invited for clinical and radiographic follow-up and were available for clinical and radiographic follow-up (follow-up rate 100%). At follow-up, the clinical examination was performed by one of the authors (TDL) not involved in the surgical treatment of the patients. External rotation and IR were assessed in flexion and in extension separately (Table II). At follow-up, we used the anterior (FADIR test) and posterior impingement test [30] to evaluate hip pain. To evaluate hip function, the Merle d’Aubigné and Postel (MDA) score [31] and full goniometric ROM were assessed (Table II). The MDA score was graded as ‘poor’ if <12, as ‘fair’ from 12–14, as ‘good’ from 15–17 and as ‘excellent’ with 18 points. Subsequent surgeries and complications were recorded using chart review (Table V). The grading of the complications was performed (Table V) according to the complication classification system for orthopaedic surgery [32]. Grade 1 complications were not included due to the retrospective study design.

**Table V.** Previous, concomitant and subsequent surgeries of the patient series are shown

	*Value (%)*
Subsequent surgeries and complications	
Hips (% of all hips) with subsequent surgeries	11 (48)
Hardware removal [hips (% of all hips)]	11 (48)
Complications requiring subsequent surgery	
Revision osteosynthesis due to delayed union (% of all hips)	1 (4)
Hip arthroscopy for adhesiolysis (% of all hips)	2 (9)
Concomitant surgeries	
Surgical hip dislocation, SHD (% of all hips)	21 (91)
Cam resection or improvement (% of all hips)	18 (78)
Anteverting PAO for acetabular retroversion (% of all hips)	5 (22)
Labrum refixation (% of all hips)	4 (17)
Acetabular rim trimming (anterosuperior, % of all hips)	4 (17)
Trimming of anterior tuberculum (% of all hips)	1 (4)
Greater trochanter trimming (anterior, % of all hips)	1 (4)
Acetabular cartilage treatment (pridie drilling, % of all hips)	2 (9)
Hip arthroscopy for adhesiolysis (% of all hips)	1 (4)
Labrum reconstruction (% of all hips)	1 (4)

All patients were asked for subjective satisfaction using the subjective hip value [33, 34] (Table II) and if they would undergo surgery again. All patients were asked to fill out questionnaires to calculate patient reported outcome measures (PROMs) at follow-up. PROMs included the University of California Los Angeles Activity (UCLA) score, the Western Ontario and McMaster Universities Osteoarthritis Index (WOMAC) and MDA score. To answer the third research question, all patients were asked if they underwent subsequent surgery.

### Statistical analysis

Normal distribution was evaluated using the Kolmogorov–Smirnov test. Statistical analysis was performed using the software Winstat software (R. Fitch Software, Bad Krozingen, Germany). Because the continuous data were not normally distributed, we only used non-parametric tests. To detect differences between the clinical ROM values pre- and postoperatively, we used the Wilcoxon-signed-rank test for continuous data and the chi-square test for binominal data.

## RESULTS

(i) The frequency of a positive anterior impingement test decreased significantly (*P* < 0.001) from preoperatively 100% to 9% (Table II). IR in 90° of flexion and in extension increased significantly (*P* < 0.001) from preoperative 10 ± 8° (0–30) to 31 ± 10° (15–50, Supplementary) and from 24 ± 11° (0–45 to 36 ± 10° (20–50, Table II). External rotation in 90° of flexion decreased significantly (*P* = 0.006) from preoperative 54 ± 18° (20–80) to 38 ± 12° (20–60, Table II).

(ii) The mean MDA score increased significantly (*P* < 0.001) from 14 ± 2 (8–15) points to 17 ± 1 (13–18, Table II, Supplementary Fig. 2) points. Subjective hip value increased significantly (*P* < 0.001) from preoperatively 33% to 77% postoperatively (Table II, Supplementary Fig. 2). Most of the patients answered that they would undergo surgery again (85%, 17 of 20 hips). Postoperative PROMs were satisfactory with a WOMAC score of 17 ± 14 (0–48) points, and the UCLA score was 6 ± 2 (3–10) points (Supplementary Table SI).

(iii) At final follow-up, there were no conversions to THA. One hip (4%, Table V) had delayed healing of the femoral osteotomy and underwent revision osteosynthesis. Implant removal was performed in 11 hips (48%, Table V). HA for adhesiolysis was performed in two hips (9%, Table V). Of the concomitant surgeries, cam resection was the most common (18 hips, 78%, Table V) and acetabular rim trimming was performed in four hips (17%, Table V).

## DISCUSSION

Variations of femoral and acetabular version [9, 17] are important features in patients evaluated for hip preservation surgery. The clinical outcome after HA for FAI [3, 12] can be influenced by both increased and decreased FV. The recent advances in the orthopaedic literature improve our understanding of FAI [15, 19]. A retrospective analysis of 23 hips of patients with anterior intra- and extra-articular subspine hip impingement and decreased FV that underwent rotational subtrochanteric femoral osteotomies to increase FV was performed. Most patients (78%) underwent concomitant cam resection and SHD (91%) for intraoperative dynamic testing of ROM and impingement and also to treat labrum or cartilage lesions. Clinical short-term outcome, subjective satisfaction and subsequent surgeries were investigated. An increased hip function, increased IR and low complication rate (Tables III and V) were found. In one patient, delayed healing of the femoral osteotomy occurred and revision osteosynthesis was performed (Table V).

A significantly (*P* < 0.001) increased IR in 90° of flexion and IR in extension (Table II, Supplementary Fig. 2) were found. Comparing the results of the current study with the literature [28, 34–37], similar results were found (Supplementary Table SII). Comparing ROM, Buly *et al*. [35] reported an increased IR in 90° of flexion from −1° to +23°. Another study [18] evaluated paediatric patients who underwent femoral rotational osteotomies, and improved IR (from 8° to 37°) was found postoperatively. This improvement of almost 30° of IR is slightly higher compared to our results.

Less hip pain and a significant decrease of the frequency of the positive anterior impingement test (Table II) were noted at follow-up in the current study. This is in line with recent reports [28, 35–37] reporting improved clinical outcomes after femoral rotational osteotomies (Supplementary Table SII). Tönnis *et al.* reported in 1991 reduced pain for 17 patients who underwent femoral rotation osteotomies [17]. In another study, a ‘marked decreased of pain’ was reported in most patients (19 out of 21) who underwent femoral rotational osteotomies [9].

In the current study, subjective satisfaction increased significantly (*P* < 0.001) from preoperatively 33% to postoperatively 77% and the mean MDA score increased significantly (*P* < 0.001, Table II, Supplementary Fig. 2). Comparing our clinical outcome with previous studies, others described 75% excellent results at a mean 6.5-year follow-up and found an improved Harris hip score for patients who underwent femoral osteotomies using an intramedullary nail [35]. Unfortunately, only a few studies reported subjective satisfaction of adult patients after this procedure [28, 34, 35, 37]. In previous studies investigating the outcome of children with cerebral palsy and increased FV, similar results were described [38, 39].

Low rate of complications was noted with one patient who underwent revision surgery (Table V). Comparing the low rate of complications to previous investigations [28, 35–37], others described a higher rate of complications such as infection, non-union or conversion to THA [35]. Kamath *et al.* [28] reported a non-union rate of 7% using the same surgical technique with femoral subtrochanteric osteotomies for treatment of abnormal FV combined with SHD. The reported non-union rate of 7% was higher compared to the current study, and this could be due to the heterogenous group of patients investigated by Kamath *et al.* [28]. No patient underwent conversion to THA in our series, while conversion to THA of 4% was described in another study with longer follow-up [35].

Based on the results of the current study, we consider femoral rotational osteotomies a safe and effective treatment to increase FV for FAI patients with decreased FV that exhibit anterior intra- and extra-articular subspine FAI. The current study differs from the previous reports for variable reasons. The main reason is that we performed patient-specific 3D simulation of hip impingement in all patients (Table IV). This is a novel method for diagnosis of extra-articular hip impingement. 3D collision detection software allowed detailed analysis of impingement location. Previously, rotational femoral osteotomies were performed mainly in adolescents and children, reporting an increase in hip IR and a decrease in external rotation [17, 18].

Routine evaluation of FV using CT or MRI to identify abnormal FV in young patients presenting with hip pain was recommended by previous studies [6]. This is in agreement with the Warwick agreement [1].

This study has limitations.

First, this retrospective case series had no control group. Second, the assessment of the clinical parameters (anterior impingement test, ROM and MDA score) was performed by different observers. This is unavoidable for a retrospective study spanning 5 years (2014–18). However, in the orthopaedic literature, substantial intraobserver agreement and interobserver agreement have been reported for these parameters [40–44] and should not affect our conclusions to a relevant degree. Third, the current series of patients reflects the initial series in which FAI patients with decreased FV and combined with other hip morphologies were treated. It seems probable that the decreased FV was not recognized initially (at the time of first surgical treatment) in some patients. To the authors, this may be an explanation for the high frequency of patients with previous HA in this series (Table I). Diagnosis and surgical decision-making to treat symptomatic anterior FAI in patients with decreased FV evolved during the study period.

## CONCLUSION

Treatment of anterior intra- and extra-articular subspine impingement resulted in decreased hip pain and increased IR. Rotational femoral osteotomies are a safe and effective treatment for young FAI patients with decreased FV. This treatment has a low complication rate and can be combined with a SHD for cam resection and/or labral refixation.

## Supplementary Material

hnad018_Supp

## Data Availability

The data underlying this article will be shared on reasonable request to the corresponding author.
