# Supplementary material for: Rotational femoral osteotomies and cam resection improve hip function and internal rotation for patients with anterior hip impingement and decreased femoral version
Source: J Hip Preserv Surg. 2023 Jul 26;11(2):85–91. doi: 10.1093/jhps/hnad018 (PMC11272641; doi:10.1093/jhps/hnad018)
Supplement: hnad018_Supp [file hnad018_supp.zip › suppl_data/Suppl Table 1 PROMs all.docx]

**Supplemental Table 1.** PROMS of the patient series are shown.

| **PROMs** | **At followup** |
| --- | --- |
| HOOS total | 67 ± 17 (39 – 97) |
| HOOS Pain | 73 ± 18 (28 – 100) |
| HOOS Symptoms and stiffness | 73 ± 15 (45 – 100) |
| HOOS Daily living | 82 ± 18 (41 – 100) |
| HOOS Sports | 60 ± 25 (13 – 100) |
| HOOS Quality of life | 47 ± 22 (6 – 100) |
| WOMAC | 38 ± 31 (0 – 101) |
| Normalized WOMAC | 17 ± 14 (0 – 48) |
| UCLA | 6 ± 2 (3 – 10) |
| HHS^13^ | 76 ± 15 (48 – 95) |
| Modified HHS | 78 ± 16 (48 – 100) |

HOOS= Hip disability and Osteoarthritis Outcome score; WOMAC = Western Ontario and McMaster Universities Osteoarthritis Index; UCLA = University of California Los Angeles Activity score; HHS = Harris hip score; Continuous values are expressed as mean ± SD and range in parentheses
