# Supplementary material for: Rotational femoral osteotomies and cam resection improve hip function and internal rotation for patients with anterior hip impingement and decreased femoral version
Source: J Hip Preserv Surg. 2023 Jul 26;11(2):85–91. doi: 10.1093/jhps/hnad018 (PMC11272641; doi:10.1093/jhps/hnad018)
Supplement: hnad018_Supp [file hnad018_supp.zip › suppl_data/Suppl table 2 Literatur table rotational osteotomies2022.docx]

**Supplementary Table 2.** Studies investigating rotational femoral osteotomies to increase femoral version

| **Author (year)** | **Followup (years)** | **No. of hips (patients)** | **Mean age (years)** | **Type and / or location of osteotomy (hips)** | **Description of results (no of hips [%])** |
| --- | --- | --- | --- | --- | --- |
| Tönnis and Heinecke (1991) | NR | 17 | (12–30) | Rotational OT | Pain was absent, movement became more relaxed, diminished femoral antetorsion syndrom |
| Tönnis et al. (1999) | 7 (2–15) | 136 (116) | 39 (19–62) | I: Rotational (21) II: Varus and rotational (25) III: Valgus and rotational (8) | overall: 113/136 (83%) with decrease of pain; I: 20/21 (95%) with decrease of pain; II: 19/25 (76%) with decrease of pain III: 8/8 (100%) with decrease of pain; 73 varus OT: 60/73 (82%) with decrease of pain 9 valgus OT: 6/9 (67%) with decrease of pain |
| Huber et al (2009) | NR | 39 (25) | 13 (9-18) | Subtrochanteric rotational osteotomy to treat reduced femoral antetorsion | All osteotomies healed without secondary displacement or angulation. Internal rotation improved from a mean of 8.6° (-5 to 20°) preoperatively to 37°(25 to 60°) postoperatively. No complication related with the femoral osteotomy. |
| Kamath et al. (2015) | NR | 28 (26) | 21 (12–43) | I: Increased FV>20°  II: Decreased FV<0° | Diagnosis included hip dysplasia, cerebral palsy, down syndrome and impingement. Subtrochanteric OT for femoral maltorsion through a surgical dislocation approach. One delayed union, one plate failure was noted. |
| Buly et al (2018) | 6.5 (2–20) | 55 (Total)  I: 39; II: 16 | 29 (14–59) | I: Derotational subtrochanteric OT (39)  II: Rotational OT (16) | The results were rated as excellent in 75%, good in 23%, and fair in 2%. The modified Harris Hip Score improved by 29 points in the remaining 52 cases.  Subsequent surgery was required in 78% of hips, 91% of which were implant removals. The osteotomies were performed closed with an intramedullary saw. |
| Mastel et al (2021) | 1.5 | 33 (29) | 29 (17-45) | Decreased FV<5°  Mean preoperative FV was -3° | 97% reported significant improvement.  The mean post-operative (iHOT-33) score of 71 points, mean pre-operative score of 42.8 points for 11 patients with available pre-operative scores.  There were three cases (9%) of delayed union and two cases (6%) of non-union early in the series. Locking screw removal was performed in 33%, overall re-operation rate 45%. |
| Hatem et al (2021) | 2 (1-6) | 37 (34)  I: 15  II: 22 | 33 (15-54) | I: Increased FV (15)  II: Decreased FV (22) | The mean mHHS improved from 58 ± 14 before PFDO to 82 ± 16 at follow-up. Improvement in the mHHS above the (MCID) was observed in 33 hips (89%). Revision procedure with a larger intramedullary nail was necessary in 2 hips to treat nonunion. |
| Rigling et al (2021) | 3 | 25 I: 18, II: 7 |  | I: Decreased FV<4° (18) II: Increased FV>28° (7) | subtrochanteric rotational osteotomy with hip arthroscopy SHV improved from 52% to 72%, and HHS from 68 to 86 points. |
| Mastel et al (2022) | I: 1.5 II: 2.5 | I 10  II: 10 | I: 36 (17-46)  II: 33 (20-44) | I: Decreased FV<5° treated with hip arthroscopy  II: Decreased FV<5° treated with femoral derotation OT | Both groups demonstrated statistically and clinically significant improvement in the post-operative (iHOT-33) scores [median improvement: FDO group, 38 points; hip arthroscopy group, 36 points. Preoperative median anteversion -0.5° for both groups. |

iHOT= International Hip Outcome Tool (iHOT-33); OT= osteotomy; MCID= minimum clinically important difference; no study with cerebral palsy patients was included
