# Supplementary material for: Rotational femoral osteotomies and cam resection improve hip function and internal rotation for patients with anterior hip impingement and decreased femoral version
Source: J Hip Preserv Surg. 2023 Jul 26;11(2):85–91. doi: 10.1093/jhps/hnad018 (PMC11272641; doi:10.1093/jhps/hnad018)
Supplement: hnad018_Supp [file hnad018_supp.zip › suppl_data/Supplemental Figure Legends.docx]

**Supplemental Figure Legends**

**Supplemental Figure 1.** Frequency of extraarticular subspine impingement based on 3D-CT for preoperative dynamic impingement simulation is shown. Anterior impingement test is performed in 90° of flexion and 30° of internal rotation.

**Supplemental Figure 2A-C.** Results of the Merle d’Aubigné score (A), subjective hip value (B) and internal rotation in 90° of flexion (C) of the patients that underwent femoral rotational osteotomy are shown.
