# Supplementary figures and images for: Rotational femoral osteotomies and cam resection improve hip function and internal rotation for patients with anterior hip impingement and decreased femoral version
Source: J Hip Preserv Surg. 2023 Jul 26;11(2):85–91. doi: 10.1093/jhps/hnad018 (PMC11272641; doi:10.1093/jhps/hnad018)

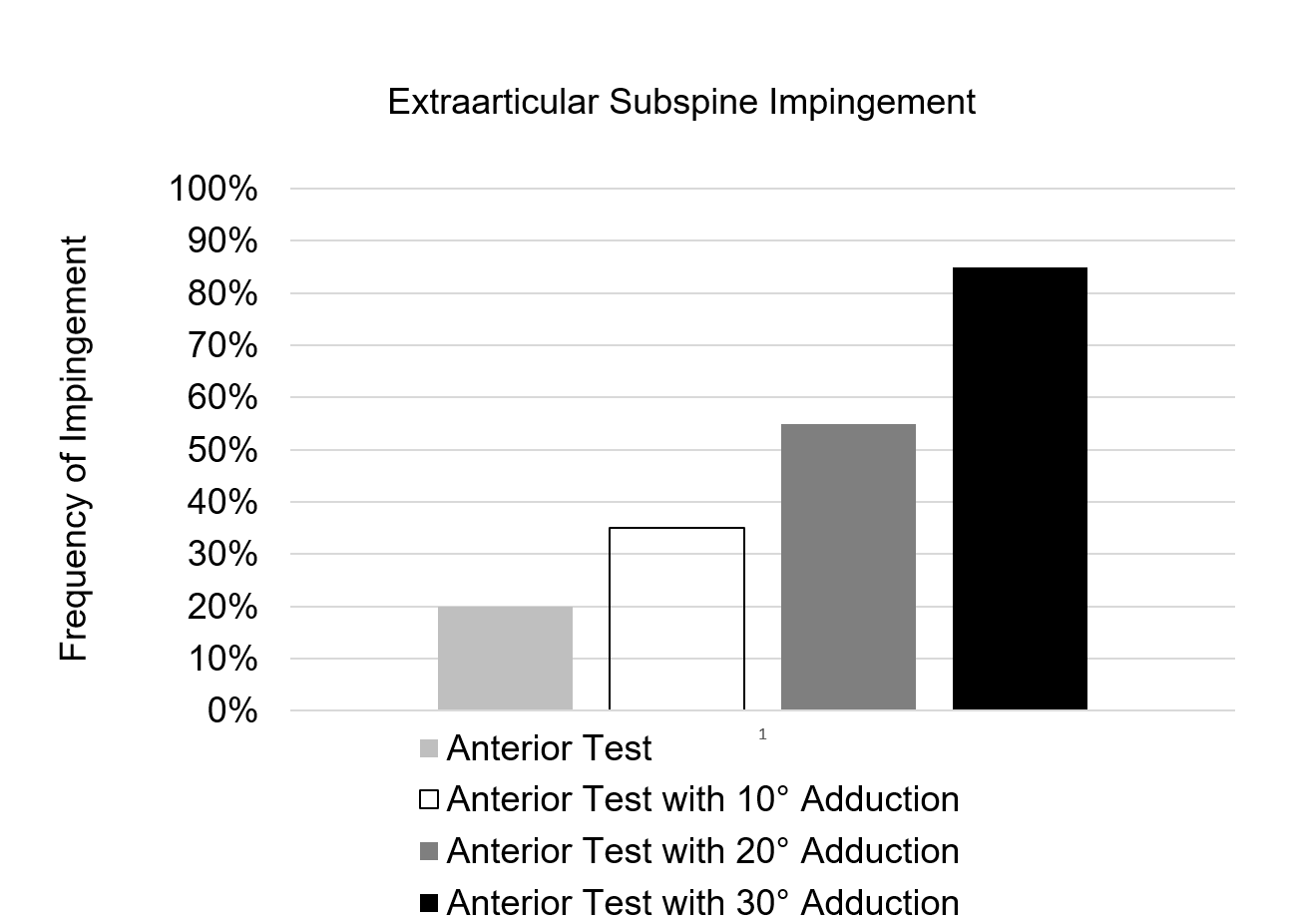

Supplement: hnad018_Supp [file hnad018_supp.zip › suppl_data/Figure_frequency_of_subspine_FAI_ Supplemental Figure 1. .PNG]

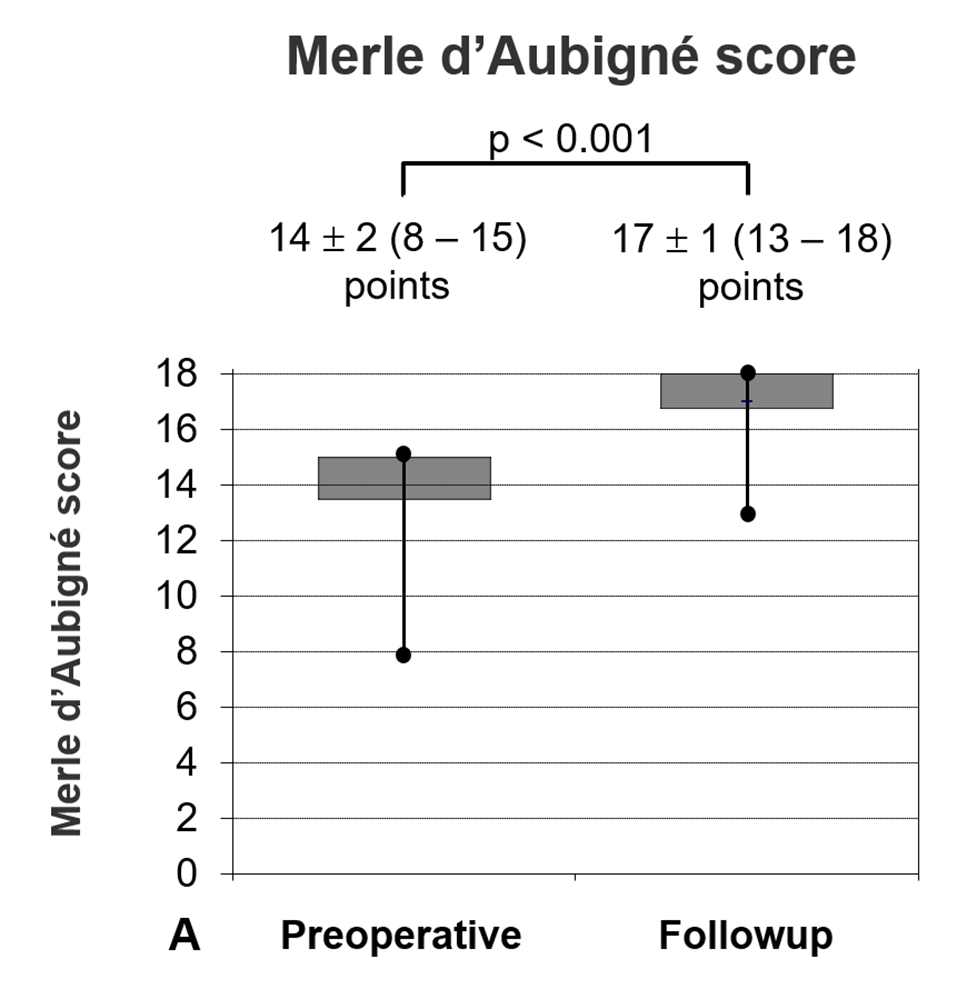

Supplement: hnad018_Supp [file hnad018_supp.zip › suppl_data/Supplemental Figure 2a.tif]

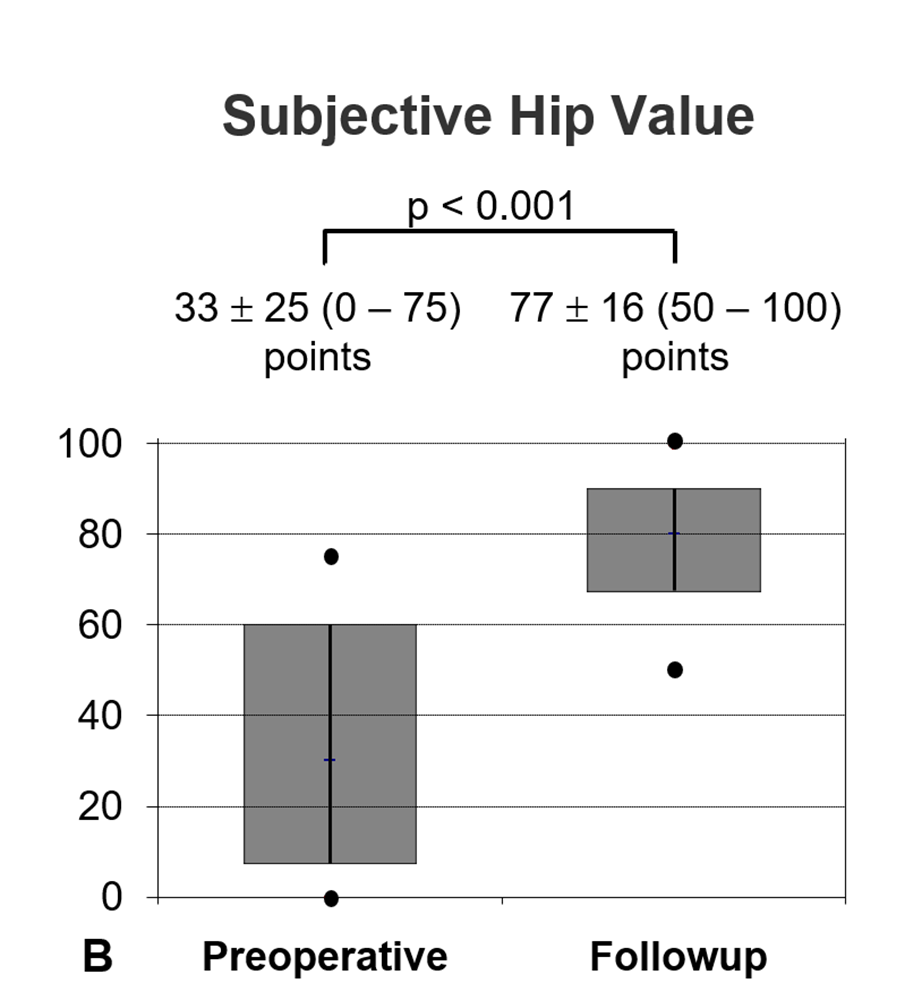

Supplement: hnad018_Supp [file hnad018_supp.zip › suppl_data/Supplemental Figure 2b.tif]

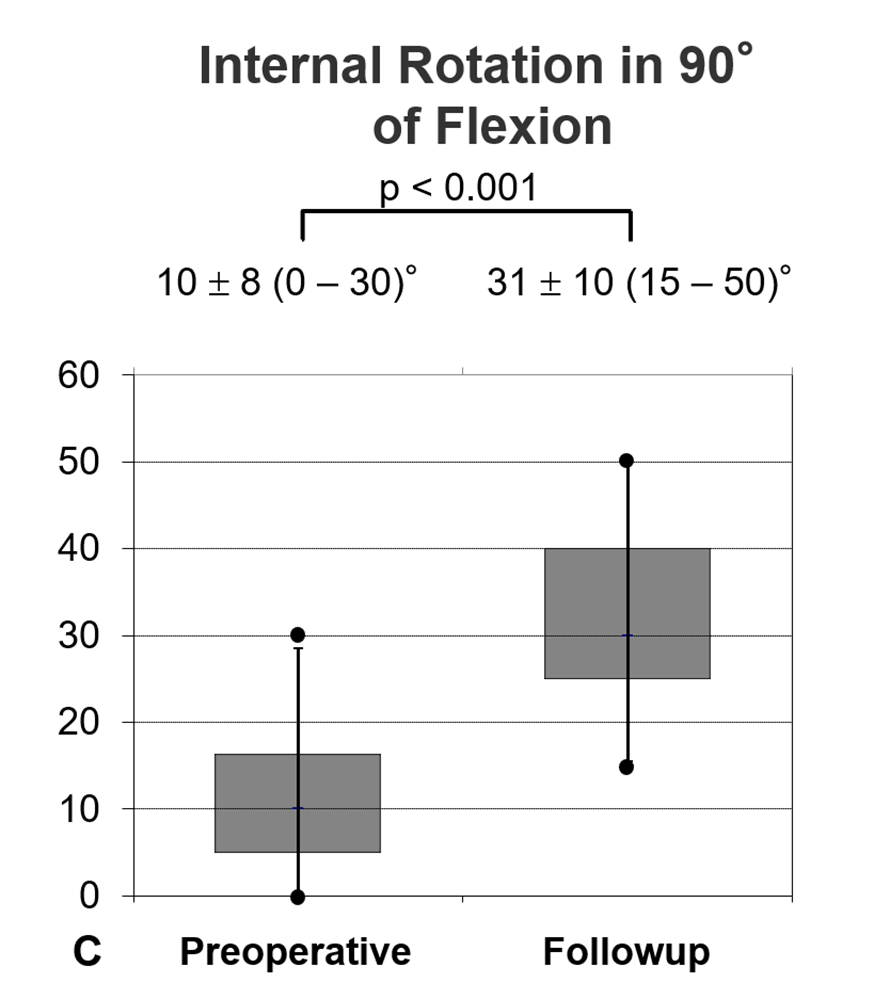

Supplement: hnad018_Supp [file hnad018_supp.zip › suppl_data/Supplemental Figure 2c.tif]
